# Emotion dysregulation, self-image and eating disorder symptoms in University Women

**DOI:** 10.1186/s40337-015-0083-x

**Published:** 2015-12-01

**Authors:** Elin Monell, Louise Högdahl, Emma Forsén Mantilla, Andreas Birgegård

**Affiliations:** Department of Clinical Neuroscience, Karolinska Institute, Norra Stationsgatan 69 7th floor, 113 64 Stockholm, Sweden; Centre for Research & Development, Uppsala University/Region Gävleborg, Uppsala, Sweden

**Keywords:** Eating disorders, Emotion dysregulation, Self-image, Mediation, DERS

## Abstract

**Background:**

We studied associations between emotion dysregulation, self-image and eating disorder (ED) symptoms in university women, and contrasted two indirect effect models to examine possible intervening mechanisms to produce ED symptoms.

**Methods:**

252 female Swedish university students completed the Difficulties in Emotion Regulation Scale (DERS), the Structural Analysis of Social Behavior (SASB) self-image measure, and the Eating Disorder Examination Questionnaire (EDE-Q). Correlations between scales were followed by five simple mediation analysis pairs with two possible pathways using five ED symptom variables as outcome. The models posited either self-image or emotion dysregulation as mediator or independent variable, respectively. ED symptoms were EDE-Q Global score, objective binge eating episodes (OBE), subjective binge eating episodes (SBE), and two variants of EDE-Q excessive exercise.

**Results:**

Emotion dysregulation and self-image were strongly correlated, and both correlated moderately with EDE-Q Global score. There were distinct indirect effects through self-image on the relationship between emotion dysregulation and ED symptoms, but not vice versa. These indirect effects were evident in relation to cognitive ED symptoms and both OBE and SBE, but not in relation to excessive exercise.

**Conclusions:**

Results suggest that even if closely related, emotion dysregulation and self-image both contribute unique knowledge in relation to ED symptoms. Self-image as an intervening mechanism between emotion dysregulation and ED symptoms is relevant for models of the development, maintenance and treatment of ED, as well as treatment focus.

## Background

Eating disorders (EDs) are relatively common among young women. A recent cohort-based study using Swedish population and healthcare registers found that adolescence is a high-risk period for the development of an ED, where the incidence of any ED by 2009 was 457.4 cases per 100,000 persons in the peak 16–17 year old age category in women [1]. A recent meta-analysis of community- and health-care based studies on prevalence of EDs found that the expected lifetime prevalence for any ED in women varies between 4.3 and 8.6 % depending on diagnosis and diagnostic criteria [2]. A comprehensive understanding of the etiology and maintenance of EDs is yet to be developed. Many researchers emphasize emotion regulation as important to move forward, although the longitudinal data needed for causal models is as yet largely lacking [3–5]. The present study related ED symptoms to emotion regulation and self-image, two factors previously shown to be related to ED when examined separately [6–8]. Briefly, emotion regulation is the ability to make sense of and manage one’s emotions, and self-image is an organizing principle guiding habitual intrapersonal behavior and interpretation of and responses in social interaction. Both emotion regulation and self-image are thought to be formed in interaction with significant others [9, 10], and both relate to social behavior and self-directed behavior, suggesting that the constructs might be interconnected or partly overlapping. Emotion regulation and self-image have been proposed to contribute to both development and maintenance of EDs [11–13]. However, the interrelationships between emotion regulation, self-image and EDs are currently unclear.

### Emotion regulation

Emotion regulation refers to the acquired ability to recognize, understand, and accept one’s emotions, as well as strategies to modulate the experience and expression of emotions in line with long-term goals and values [14]. This ability develops over time, and early childhood interactions with caregivers seem to be of great importance [10, 15]. Emotion dysregulation is suggested to be a central factor in the development and maintenance of various problematic behaviors like self-harm and violence towards others, where the most comprehensive theoretical work is Linehan’s bio-psychosocial model of emotion dysregulation in borderline personality disorder (BPD) [16]. This model has also been used to describe similar processes in anorexia nervosa (AN) [3]. The model describes the development of emotion dysregulation as a transactional process between individual emotional vulnerabilities and invalidating responses from the social and family environment [15, 17]. Individual emotional vulnerability consists of relatively stable influences of temperamental affective tendencies (e.g. emotional sensitivity, reactivity, and time needed to recover from emotional events) as well as more transient factors related to sleeping habits, diet, physical health, etc. Invalidating response relates to interpersonal interactions where an individual’s emotional and cognitive experiences are overlooked, misunderstood, or criticized by others. A vicious circle of vulnerability and invalidation where increased emotional arousal, and ensuing increased difficulty to accurately communicate emotional states, then risks maintaining and reinforcing emotion dysregulation and usage of dysfunctional regulatory strategies, for example self-harm in BPD patients [3, 15]. In sum, unfortunate transactional processes over time are thought to create pervasive trait-like patterns of emotion dysregulation. In everyday life, high intensity and/or long duration of emotional arousal tend to heighten the risk of emotion dysregulation in response to everyday emotional events [3].

### Emotion dysregulation and EDs

The ability to identify and describe emotions is decreased among women with EDs [4, 18]. There is evidence for the BPD model of emotion dysregulation in AN, with respect to both individual vulnerabilities such as emotional sensitivity and reactivity, as well as experiences of invalidating response [3]. Women with EDs are also more likely to use dysfunctional regulation strategies such as rumination and suppression in response to negative affect [11, 19]. Fairburn, Zafran and Cooper suggest an inability to cope with intense emotional states as a transdiagnostic feature in some ED-patients and also state that ED behaviors may serve as maladaptive forms of emotion regulation, comparable to the use of self-harm in BPD [20]. The most widespread theory on the function of ED-behaviors suggests that binge eating, with or without subsequent purging, provides distraction from or amelioration of painful inner states, negatively reinforcing the behavior [20, 21]. In line with this theory, several experimental studies have found that negative mood precedes episodes of binge eating among women with bulimia nervosa (BN) [22, 23], binge eating disorder (BED) [24–26], and non-clinical women [27], even though the ameliorative functon of binge eating is yet to be consistently experimentally shown [28]. There is some support that restricting behaviors and excessive exercise may serve a similar function as suggested for binge eating [29, 30].

However, the relation between emotion dysregulation and EDs is still unclear, partly since conceptualizations and measurements of emotion dysregulation have differed in previous research. A growing number of ED researchers have used the Difficulties in Emotion Regulation Scale (DERS), measuring difficulties in awareness, acceptance and understanding of emotions, as well as lack of strategies to manage emotions, control impulses and problems engaging in goal-directed behavior while in distress. Recent studies using the DERS show that for women with either AN or BN, more ED symptoms was significantly correlated with more emotion dysregulation [6, 31]. Other studies show that individuals with AN or BN have significantly more difficulties with all DERS aspects compared to healthy comparison groups [7, 32, 33], and that higher DERS scores (i.e. more emotion dysregulation) after AN treatment predicted maintenance of AN psychopathology over time [34]. In addition, more ED symptoms were related to higher DERS scores in an ED group, a healthy comparison group, and two psychiatric groups [12], as well as in groups of healthy young women [35, 36].

### Emotion regulation and self-image

Interpersonal factors that impact emotion regulation are increasingly in focus in ED research. For example, two recent meta-studies found that aspects of interpersonal difficulties affecting emotion regulation may serve as maintaining factors for EDs, these difficulties including for example the avoidance of expressing feelings to others, interpersonal distrust, more negative interactions with others, insecure attachment and perceived social inferiority [18, 37]. As indicated, the term “interpersonal” includes not only patterns of interaction with others, but also intrapsychic experiences related to self and others. A fruitful approach, not utilized in previous research, may then be to study emotion regulation within an interpersonal framework, drawing on theory concerning how the sense of self is formed and maintained in a social context. Such theoretical approaches can be found in the field of interpersonal theory, where self-image is a central concept [38].

Self-image is mainly described as self-directed behaviour, i.e. how an individual treats him-/herself. These behaviours will have cognitive, emotional and social implications since the self-image will guide how interactions with others are perceived and interpreted by the individual [39]. Further, people tend to behave in ways that evokes responses from others that are in line with the self-image, leading to its confirmation and preservation [9]. According to theory, the self-image is developed over time when patterns of social interaction are introjected, i.e. the way others (especially attachment figures) treat you will model your future self-treatment [9]. An individual’s emotional vulnerabilities, as noted from theories on emotion regulation, will affect others’ responses. This may in turn affect the sense of self as capable or deficient, as worthy of care, encouragement, criticism, or neglect. This self-view will likely impact emotional experiences and their expression, leading to a transactional and mutually reinforcing development of emotion regulation strategies and self-image, in an interpersonal context.

### Self-image and EDs

Self-evaluation, especially measured as self-esteem, in ED patients has been studied before where ED patients frequently evaluate themselves more negatively than healthy comparison groups [18]. Other aspects of self-evaluation studied in relation to EDs are for example self-efficacy, self-directedness and self concept [40, 41]. The Structural Analysis of Social Behavior (SASB) is a model of self-image (and social behavior) based on interpersonal theory [42]. According to the SASB, the self-image has both trait and state aspects: there is considerable stability in self-image and evidence of associations with relationships to early significant others, but there is also variability in response to current stressors and situations [43–45], as exemplified by the fact that self-report is sometimes used with instructions to rate the self-image “at best” and “at worst” [46]. The SASB self-image thus differs from other self-related concepts in going beyond self-evaluation and self-directed feelings, to also include self-directed behavior and the implications of self-image for social behavior.

The SASB model (and its set of measurements) organize self-image in a circumplex with two dimensions: horizontal *Affiliation* axis ranging from self-love to self-attack and vertical *Autonomy* axis from enmeshment to differentiation. A positive self-image (predominantly self-love) is characterized by self-affirmation, self-love and self-protection whereas a negative self-image (predominantly self-attack) is characterized by self-blame, self-attack and self-neglect.

ED research using the SASB has found that patients with an ED have a more negative self-image compared to healthy and subclinically depressed comparison groups [8]. Initial SASB self-attack among ED patients further predicted treatment outcome after 3 years, being a stronger predictor than initial ED symptoms, general psychopathology, interpersonal relationships, and occupational status [47]. Specific self-image aspects also predicted outcome in different ED diagnoses [13] as well as treatment dropout [48]. Specific self-image aspects relate much more strongly to ED symptoms in young adolescent ED patients than in healthy young adolescents, a pattern also evident in older female adolescents and young women (the latter result was partly based on the same sample as the present study) [49, 50]. Also, relevant for outcomes relating to emotion dysregulation research, self-image has shown associations with suicidal behavior in ED patients [51].

### Aim

In summary, interactions with significant others are an important way to acquire emotion regulation strategies, while at the same time, emotion regulation affects how such interactions occur. Interactions with others over time also model self-image, defined as internal self-directed behaviour, which from a here-and-now perspective has cognitive, emotional and social implications. Previous research has found significant connections between EDs and both emotion dysregulation and self-image when examined separately. As described, both emotion dysregulation and self-image develop over time starting in early childhood, with likely intertwined developmental paths. They may be risk factors for later ED development, and may impact ED symptoms in the present by emotion regulation affecting one’s sense of self, which may impact ED symptoms, or the sense of self may affect emotion regulation, which in turn impacts ED symptoms.

No previous research has examined the association between self-image and emotion dysregulation. The aim of the present study was to do this and to associate both concepts to ED symptoms. We aimed to investigate which theoretical model best fits the data by contrasting two possible models to evaluate indirect effects (mediation): self-image as a mechanism for emotion regulation, or emotion regulation as a mechanism for self-image, to produce each of five different types of ED symptoms. Knowledge in this area may inform prevention and etiological models by suggesting hypotheses concerning mechanisms of vulnerability and how they are expressed during development, and what symptoms are likely to ensue. Also, findings may have implications for treatment efforts, by identifying more proximal and distal intervention targets to ameliorate symptoms.

## Method

### Participants

The sample consisted of 252 female Swedish university students with a mean age of 23.7 years (*SD* 3.58, range 19–35) and a mean BMI of 22.4 (*SD* 3.68, range 15.6–44.4). 374 students were given questionnaires whereof 288 (77 %) completed participation (i.e. returned the completed questionnaires). Thirty-six of these (12.5 %) were excluded prior to analysis: eight due to missing data (one without background information, seven with missing data for too many items on single instruments) and 28 due to age >35 years (this maximum age was decided *a priori* to match the sample to typical clinical ED populations). No final participant had more than two missing items for any one instrument. Since variables from the relevant measures could be computed with some missing items no imputation of data was conducted. Of the final sample, 181 (71.8 %) participants were recruited at lectures, 34 (13.5 %) at fixed occasions for drop in participation, and 37 (14.7 %) by advertisement around the campus area. There were no significant differences between subsamples depending on recruitment method on any variable (all ANOVA and post hoc *p*’s > .05, data not shown).

### Instruments

*Eating Disorder Examination Questionnaire (EDE-Q, version 4.0)* was used to assess ED symptoms [52]. The EDE-Q contains 36 items focused on the past 28 days and provides a Global score, four subscales (Eating concern, Shape concern, Weight concern, Restraint), and information regarding ED behavior. The Global score, where higher scores indicate more severe eating pathology, is used as an outcome for cognitive ED symptoms. The other outcomes were the following ED behavior items: presence/absence of objective binge eating episodes (OBEs), subjective binge eating episodes (SBEs), and excessive exercise. OBEs are defined as reporting rapidly eating objectively large amounts of food combined with loss of control over eating. SBEs are defined as loss of control but not eating large amounts. Excessive exercise is defined as intense exercise with the purpose to control weight or shape. We included both presence/absence of excessive exercise, as well as frequency of excessive exercise ≥ twice/week corresponding to the diagnostic cut-off in DSM-IV for BN. The latter was included in order to test exercise at diagnostic criterion level since any episodes of excessive exercise was reported by a substantial proportion of participants. We did not include vomiting as an outcome since only 11 participants (4.4 %) reported this behavior, and even fewer reported diuretic and laxative use (four participants for each). The EDE-Q has good psychometric properties [53], with satisfactory concurrent validity [52, 54], acceptable temporal stability and acceptable internal consistency [53, 55]. The Swedish version of the EDE-Q has shown satisfactory validity and acceptable reliability [56]. Mean Cronbach’s α for the EDE-Q subscales in the present sample was .84 (range .81–.91).

*Difficulties in Emotion Regulation Scale (DERS)* consists of 36 items measuring aspects of emotion dysregulation, and provides a Total score and six subscales (Non-acceptance, Goals, Impulse, Awareness, Strategies, Clarity) [14]. The Total score, where higher scores indicate more difficulties with emotion regulation, is used in the statistical analysis. In its original form, the items in each subscale are summed to calculate the scores and the subscale scores are then summed to calculate the Total score. Since the subscales consist of different numbers of items and sums are therefore difficult to compare, we used mean scores that are comparable and interpretable in terms of the response scale metric, and the Total score was calculated as the average of the subscale scores. The DERS has shown good internal consistency and good test-retest reliability [14]. The DERS was translated from English to Swedish for the present study, using two independent translators (authors EM and AB) as well as back-translation from Swedish to English by a native English speaker. Preliminary factor analysis results for the current sample fairly reproduced the six-factor structure of the original (Birgegard: Factor structure of a Swedish version of the Difficulties in Emotion Regulation Scale, in preparation). Mean Cronbach’s α for the DERS subscales in the present sample was .84 (range .75–.90).

*Structural Analysis of Social Behavior introject, Swedish version 2.0 (SASB intrex version 3*^*rd*^*surface, self-image)* was used to assess self-image [57]. The SASB introject consists of 36 items operationalizing self-directed behavior and attitudes. Responses form eight variables (clusters), six of which form the Affiliation score (by weighting the variables according to their proximity to the horizontal axis and dividing by the sum of the weights). SASB Affiliation is used in the statistical analysis and ranges from −100 to 100 where scores below zero indicate more self-directed attack and scores above zero indicate more self-directed love. The English language version of SASB intrex has shown good reliability and internal consistency [57]. The Swedish version of SASB intrex is highly consistent with the American version (Armelius et al., unpublished manuscript, 1993) and has shown good internal consistency (Armelius, unpublished manuscript, 2001). Mean Cronbach’s α for variables used to compute the SASB Affiliation score in the present sample was .78, range .65–.86.

### Procedure

Questionnaires were administrated as a booklet in the following order: informed consent and contact details, questions about age, height, and weight, SASB, DERS and EDE-Q followed by four additional questionnaires not included in the present study. Time for participation was estimated to 30–40 min. Participants who were recruited at lectures got the booklet and a postage paid envelope, those who responded to advertisements emailed their address and were sent the materials, and recruitment information about the study was equated. Occasions for drop in on-site participation (at a university department) were announced by bulk email and on notice boards. All participants gave informed consent regarding storage and use of data, and were rewarded by gift certificate (approx. 15 USD) or course credit. The study was approved by the Stockholm Regional Ethics Review board (2013/243-31/3).

### Statistical analysis

Statistical analyses were performed in SPSS Statistics version 21.0 for Mac. Scale correlations were Pearson *r* for continuous measures, point biserial coefficients (*r*_*pb*_) when one variable was dichotomous, and the phi coefficient (*r*_*φ*_) for two dichotomous variables, all interpreted the same. Simple mediation analysis with two possible pathways was conducted to evaluate indirect effects, using the PROCESS macro for SPSS by Hayes, Model 4 (mediation of independent variable X on outcome Y by mediator M) [58]. Following guidelines provided by Hayes, all mediation analyses were conducted using unstandardized variables. Therefore the pathway coefficients calculated by PROCESS are expressed in the metric of the variables. The first pathway posited DERS Total score as X and SASB Affiliation as M, and the second pathway did the opposite. Five model pairs with five different (ED-related) outcomes (Y) were tested: 1) EDE-Q Global score (EDE-Q), 2) presence/absence of OBEs, 3) presence/absence of SBEs, 4) excessive exercise ≥ twice/week (Regular EE), and 5) any excessive exercise (Any EE). The first outcome, EDE-Q Global score, was continuous and the other four were dichotomous. PROCESS is based on ordinary least squares regression for mediation models with continuous outcomes and logistic regression for dichotomous outcomes. Statistical inference for potential indirect effects was conducted through bias-corrected bootstrap confidence intervals based on 10.000 bootstrap samples [59]. The effect size for indirect effect (mediation path) for the continuous outcome model was Preacher and Kelley’s Kappa-squared (*κ*^2^) [60], ranging from 0 to 1, where small ≥ .01, moderate ≥ .09, and large effect size ≥ .25 conventions are applicable. Effect sizes for indirect effects with dichotomous outcomes are not available.

## Results

### Sample characteristics and scale correlations

Correlations between measures used in the indirect effect (mediation) models are shown in Table 1. Emotion dysregulation and self-image were strongly negatively correlated, and both correlated moderately with cognitive ED symptoms (EDE-Q Global score). All DERS subscales were also significantly correlated to EDE-Q Global Score (*r =* .117 to .369, not shown in Table 1). For the behavior-related ED variables, 47 (18.7 %) of all participants reported objective binge eating episodes, 61 (24.2 %) reported subjective binge eating episodes, 47 (18.7 %) reported regular excessive exercise, and 90 (35.7 %) reported any excessive exercise. All behavior-related ED variables correlated significantly with EDE-Q Global Score, but only the binge eating variables correlated significantly with emotion dysregulation and self-image. Since a significant correlation is not required for mediation analysis according to recent literature [61], all were tested for indirect effects.

**Table 1:** Descriptive statistics and Pearson correlations (r for 1–3, *r*_*ob*_ for 1–3 vs. 4–6, and *r*_*φ*_ for 4–6) and correlations (all including 4–7) among emotion dysregulation, self-image and eating disorder symptom variables

	M (SD); range	EDE-Q	DERS.	SASB	OBEs	SBEs	Reg. EE
EDE-Q	1.65 (1.21); 0–5.32	–					
DERS	2.32 (.58); 1.01–4.01	.391^***^	–				
SASB	41.93 (29.45); −47.3–96.8	−.462^***^	−.717^***^	–			
OBEs	–	.486^***^	.257^***^	−.317^***^	–		
SBEs	–	.532^***^	.212^**^	−.266^***^	.441^***^	–	
Reg. EE	–	.290^***^	.027	−.036	.029	.130^*^	–
Any EE	–	.323^***^	.096	−.087	.071	.178^**^	.642^***^

*EDE-Q* Eating Disorder Examination Questionnaire Global Score; *DERS* Difficulties in Emotion Regulation Scale Total score; *SASB* Structural Analysis of Social Behavior Affiliation Score; *OBE* Objective Binge eating Episodes; *SBE* Subjective Binge eating Episodes; *Reg. EE* Regular Excessive Exercise; *Any EE* Any Excessive Exercise

Note: ^*^ = *p* < .05, ^**^ = *p* < .01, ^***^ = *p* < .001

### Indirect effect (mediation) models

**Fig. 1:**
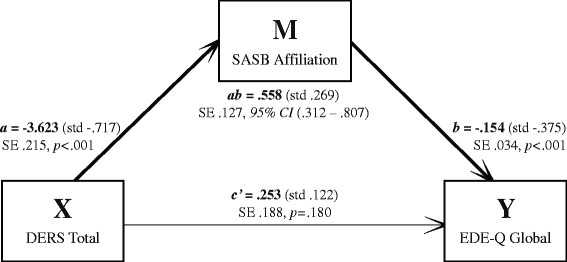
Unstandardized and standardized coefficients mediation model 1; DERS Total score as independent (X), SASB Affiliation score as mediator (M), and EDE-Q Global score as dependent (Y). N = 252. SE = Standard Error. 95 % CI = 95 % Confidence Interval

**Fig. 2:**
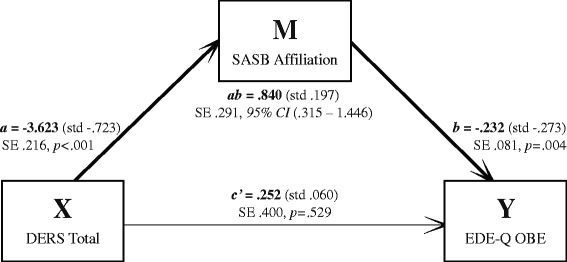
Unstandardized and standardized coefficients mediation model 2; DERS Total score as independent (X), SASB Affiliation score as mediator (M), and EDE-Q Objective Binge eating Episodes as dependent (Y). N = 248. SE = Standard Error. 95 % CI = 95 % Confidence Interval

**Fig. 3:**
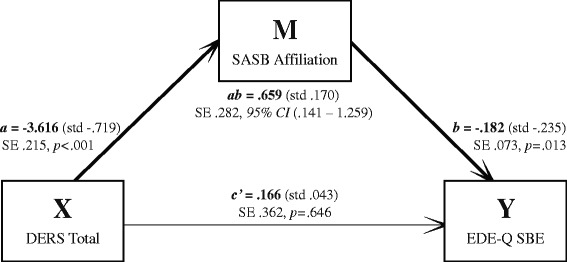
Unstandardized and standardized coefficients mediation model 3; DERS Total score as independent (X), SASB Affiliation score as mediator (M), and EDE-Q Subjective Binge eating Episodes as dependent (Y). N = 248. SE = Standard Error. 95 % CI = 95 % Confidence Interval

Results for the indirect effect (mediation) models are reported in unstandardized metric. The first pathway (where emotion dysregulation as X indirectly influenced the five different ED-variables as Y through its effect on self-image as M) had bias-corrected bootstrap confidence intervals for the indirect effect entirely above zero for three models (Table 2). Those models were the ones with outcome 1) EDE-Q Global score, 2) OBE, and 3) SBE. Coefficients are presented in Figs. 1, 2 and 3, where all unstandardized coefficients are expressed in the metric of the dependent variable, except for path *a,* which is expressed in the metric of the mediator. Standardized coefficients computed by regression analysis are presented in parentheses alongside their unstandardized counterparts in the figures.

**Table 2:** Mediation models summary: DERS as independent (X), SASB as mediator (M), and five different ED-related measures as dependent variables (outcomes; Y)

Outcome variable	Total effect (*c*)	SE	*p*	Direct effect (*c’*)	SE	*p*	Indirect effect (*ab*)	SE	95 % CIs
EDE-Q Global score (EDE-Q)	.810	.128	<.001	.253	.188	.180	.558	.127	.312 to .807
Objective binge eating episodes (OBE)	1.082	.278	<.001	.252	.400	.529	.840	.291	.315 to 1.448
Subjective binge eating episodes (SBE)	.822	.252	.001	.166	.362	.646	.659	.282	.141 to 1.259
Regular excessive exercise (Reg. EE)	.117	.275	.670	.007	.396	.985	.109	.260	−.391 to .626
Any excessive exercise (Any EE)	.343	.225	.128	.248	.323	.444	.095	.228	−.342 to .563

All coefficients are unstandardized and expressed in the metric of the outcome variable

CIs = bias-corrected bootstrapped confidence intervals based on 10000 bootstrap samples

For the model with outcome 1 (Fig. 1), the more emotion dysregulation, the less positive self-image, and the higher degree of ED symptoms. There was a moderate indirect effect of emotion dysregulation through self-image on ED symptoms (indirect effect, path *ab*: *κ*^2^ = .204), but emotion dysregulation did not influence degree of ED symptoms independent of its effect on self-image (direct effect, path *c’*). For models with outcomes 2 (Fig. 2) and 3 (Fig. 3), emotion dysregulation indirectly influenced both OBE and SBE through its effect on self-image. Again, emotion dysregulation did not significantly influence OBE or SBE episodes independent of its effect on self-image.

The bias-corrected bootstrap confidence intervals for the indirect effect of the models with outcome 4) Regular EE, and 5) Any EE both included zero (−.391 to .626 and -.342 to .563, respectively) and thus were not supported. Similarly, all indirect effect confidence intervals for models positing self-image as independent (X) and emotion dysregulation as mediator (M) included zero (Table 3).

**Table 3:** Alternate mediation models summary: SASB as independent variable (X), DERS as mediator (M), and five different ED-related measures as dependent variables (outcomes; Y)

Outcome variable	Total effect (*c*)	SE	*p*	Direct effect (*c’*)	SE	*p*	Indirect effect (*ab*)	SE	95 % CIs
EDE-Q Global score (EDE-Q)	−.190	.023	<.001	−.154	.033	<.001	−.036	.027	−.091 to .013
Objective binge eating episodes (OBE)	−.269	.058	<.001	−.232	.081	.004	−.036	.056	−.148 to .072
Subjective binge eating episodes (SBE)	−.206	.051	<.001	−.182	.073	.013	−.024	.054	−.133 to .081
Regular excessive exercise (Reg. EE)	−.031	.054	.565	−.030	.078	.699	−.001	.052	−.101 to .105
Any excessive exercise (Any EE)	−.061	.045	.168	−.026	.064	.682	−.035	.046	−.127 to .055

All coefficients are unstandardized and expressed in the metric of the outcome variable

CIs = bias-corrected bootstrapped confidence intervals based on 10000 bootstrap samples

## Discussion

The study examined the association between self-image, emotion dysregulation, and ED symptoms, and investigated which theoretical model best fits the data by contrasting two possible indirect effect (mediation) models. We found significant correlations between ED symptoms and emotion dysregulation and negative self-image, respectively. Emotion dysregulation was also highly correlated with self-image, suggesting that these constructs are closely related. The results however suggested that both contribute unique knowledge in relation to ED symptoms: there were distinct indirect effects through self-image on the relationship between emotion dysregulation and ED symptoms in university women. No direct effect of emotion dysregulation on ED symptoms was found, suggesting that emotion dysregulation has its effect via self-image as an intervening variable. As for the alternate model, no indirect effect through emotion dysregulation was found for the relationship between self-image and ED symptoms.

The indirect effects were evident for cognitive ED symptoms and for presence/absence of both objective and subjective binge eating, but not for excessive exercise. This may be because exercise reported in this sample might be related more to positive and healthy motivations, not being as symptomatic as the much less frequent binge eating. Although the exercise item asks about intense exercise with the purpose to control weight or shape, exercise responses may have a different quality in a non-clinical group than in clinical samples. Although significant, correlations between both exercise variables and EDE-Q Global score were weaker than those of the binge-eating variables. Binge eating as measured here therefore may be a more disordered behavior, and thus more representative of ED symptoms in this sample. As noted, we could not however test purging due to relative rarity of the behavior. It would be important therefore to examine possible indirect effects in relation to these behaviors in a clinical ED sample, where purging is more frequent and exercise may be more compulsive and symptomatic in nature.

The significant relationship between ED symptoms and emotion dysregulation measured by the DERS are in line with previous research in both clinical and healthy samples [7, 33, 36]. Higher levels of emotion dysregulation as related to more ED symptoms seem to be a robust finding across several studies including this one. There is also a large body of research relating aspects of emotion dysregulation, such as reduced ability to identify and describe emotions and usage of dysfunctional strategies to regulate emotions, to the presence of ED symptoms [3–5, 11]. In relation to the theories on ED behavior, in particular binge eating, as maladaptive emotion regulation strategies [20, 21], our results did show significant, although rather weak, relationships between emotion dysregulation and binge eating. Lastly, there are hypotheses that emotion dysregulation may both precede and maintain ED [3, 11, 19]. Our results, being based on self-reported cross-sectional data, cannot clarify causality but are consistent with emotion dysregulation as important for ED symptoms, even in this non-clinical sample.

The significant relationship between ED symptoms and self-image measured by the SASB is also in line with previous research, where a more negative self-image was associated with more ED symptomatology in both clinical and healthy samples of different ages [8, 49, 50]. Our result of negative self-image being related to more ED symptoms is also in line with results from studies using related but less complex measures of different types of self-evaluation [18, 40]. In a broader perspective, our results on ED symptoms and self-image, being a concept of interpersonal origin, are also consistent with the notion that interpersonal factors are relevant for development and expression of ED [18, 37].

As for the conceptual and theoretical similarities between emotion dysregulation and self-image presented in the introduction [9, 18, 37, 39], the strong significant correlation between the DERS and the SASB indicates that they are indeed closely related. The findings regarding indirect effects may further suggest a hypothesis that emotion regulation is a more basic vulnerability factor that affects self-treatment. Although these factors are likely to evolve interactively, emotion regulation may be a lower-level set of skills and self-image a higher-level set of conceptions of the worth and capabilities of oneself as a person, analogous perhaps to how throwing a ball relies on individual muscles in the arm and torso, but whose subjective relevance relates to length and accuracy of the throw.

Taken at face value, our findings indicate that a young woman with difficulties regulating emotions is vulnerable to ED symptoms if those difficulties negatively impact her self-image, but that emotion dysregulation per se will not lead to ED symptoms. For example, intense disappointment or feelings of rejection may lead to negative self-appraisal concerning weight and shape, and possibly binge eating as a problematic means of regulating the emotion, if it arises in the context of habitual self-blame, self-attack and lack of self-affirmation. If such factors are not present, our findings indicate that ED symptoms are less likely to occur. Importantly also, the SASB self-image measure operationalizes self-directed behavior, and not merely evaluations, attitudes or cognitions. Thus, ED symptoms may be construed, given our data, as negative self-treatment arising in the context of negative emotion that cannot be regulated adaptively. Besides affecting the individual, such a process will most likely have interpersonal consequences. Building on the transactional nature of emotion regulation proposed by Linehan [16], ED symptoms are unlikely to be met by others as an accurate expression of the emotion that triggered them. Instead, others may signal worry, frustration, or irritation (i.e. invalidating responses). This might give rise to feelings of guilt and alienation that, building on interpersonal theory [9, 39], could confirm a negative self-image. These kinds of processes could contribute to the increased social vulnerability experienced by individuals with EDs [18, 37], as well as exacerbating the risk of negative emotional arousal in new situations [3]. While broadly consistent with developmental models of psychopathology stressing interactions between temperamental traits (e.g. emotional vulnerability) and relational processes [3, 15, 17], this description is speculative and requires further refinement and research.

### Implications

Our findings may indicate targets for treatment and prevention of ED. The indirect effects suggest for example that emotion regulation training, suggested as an important treatment intervention [31, 32, 34], needs to (also) target self-image, i.e. habitual self-directed behavior and the impact of difficult situations on patients’ conceptions of themselves as individuals. Our findings posit self-image as the more proximal construct in relation to presenting ED symptoms. Therefore, the relationship of self-image to body dissatisfaction, control of food intake, and compensatory behavior may be clearer to the patients themselves. On such a shared understanding and alliance basis, investigating the influence of negative emotion, and explicit emotion regulation skills training, may be more successful. Continuing the ball-throwing analogy, if a person consistently misses the target it may become relevant to train individual muscles, but such coaching must be based on, and continually return to, the higher-level synthetic ability to hit the mark. In addition, to prevent confirmation of the patient’s problematic self-image by processes described by Benjamin [9], and thereby risk maintaining both ED symptoms and emotion dysregulation, the therapist may profitably consider interpersonal interactions both within and outside the therapeutic relationship from this perspective.

In addition to implications for treatment, our results points to the importance of attending to emotion regulation and self-image as a way of preventing ED. The ability to recognize, understand, and accept one’s emotions, as well as having adaptive strategies to modulate the experience and expression of emotions, might be protective against negative self-treatment and possible subsequent ED symptoms. Also, prevention efforts may profit from explicitly addressing how problems handling emotions translate into self-treatment. For example, offering alternatives to subjective contingencies such as “I always react hysterically to things and ruminate on them, I’m a stupid and useless person” could prevent further translation into perfectionistic or impulsive attempts to deal with anxiety. A general awareness of the transactional associations between emotions, self-directed behavior and interpersonal interactions should be helpful [9, 16]. This might be of special importance among professionals encountering young individuals, for example in primary care, student health services, or family counseling. On a basic level, individuals can be informed on how to decrease the risk of too intense emotional arousal by properly managing sleep, diet, and physical health [3, 16]. For those who have been caught in vicious interactional patterns and dysfunctional ways of managing emotions, a greater understanding and clarification of the processes involved may be helpful. The presence of ED symptoms, i.e. negative self-treatment arising in the context of dysregulated negative emotion, puts the focus on improving social interactions and strengthening positive self-treatment as an important adjunct to development of skills to tolerate and manage a wide range of emotions.

### Strengths and limitations

The results of the study are based on data from well-established and clinically relevant scales; the EDE-Q, the DERS and the SASB, all previously used in both ED research as well as research on various forms of psychopathology. Response rate was fair, and psychometric properties of the scales in our sample were generally acceptable to excellent. However, we had only cross-sectional data and cannot infer causality. Also, while response rate was fair, no attrition analyses could be performed, and it is possible that those who participated were systematically different from those who chose not to. Cronbach’s α for one the six subscales forming SASB Affiliation was relatively low (.65), possibly attenuating associations with other variables (although the strong *r* vs. the DERS may speak against this). However, the remaining subscales all had acceptable internal consistency (Cronbach’s α >.79), suggesting that the full Affiliation variable was reasonably internally consistent. The DERS was translated from English to Swedish for the present study, leaving the Swedish version of DERS so far relatively untested. However, preliminary psychometric investigation (including factor structure) suggests satisfactory properties (Birgegard: Factor structure of a Swedish version of the Difficulties in Emotion Regulation Scale, in preparation). We also ran a large number of analyses, leading to Type I error risk. This coupled with the fact that the present study is the first on the included interrelationships underlines the need for cautious interpretation and future replication. Of note however, *p* values were mostly substantially below .05 and the pattern of results was fairly consistent (e.g. exercise variables were not implicated overall but cognitive and binge-related ED symptoms were, and indirect effects was clearly found in one direction but not the other), suggesting some reliability in our findings. Nonetheless, given the complexity of the theoretical background, the aim of this study as well as the chosen methods, general caution is warranted for both interpretation and possible implications of the results.

## Conclusion

The present study suggests that emotion dysregulation and self-image are closely related but both contribute unique knowledge in relation to ED symptoms: emotion dysregulation may contribute to ED symptoms via the self-image. This finding expands current knowledge on the relationship between emotion dysregulation and ED symptoms, with relevance for theories on the development and maintenance of ED, and may also contribute to more effective ED treatments. For the latter, further research on clinical samples is important, and to accurately infer causality between the constructs, experimental or longitudinal/sequential data are needed.

## Abbreviations

ED: eating disorder
AN: anorexia nervosa
BN: bulimia nervosa
BED: binge eating disorder
DERS: Difficulties in Emotion Regulation Scale
SASB: Structural Analysis of Social Behavior
EDE-Q: Eating Disorder Examination Questionnaire
OBE: objective binge eating episodes
SBE: subjective binge eating episodes
EE: excessive exercise
